# Prevented cases of neural tube defects and cost savings after folic acid fortification of flour in Brazil

**DOI:** 10.1371/journal.pone.0281077

**Published:** 2023-02-22

**Authors:** Viviane Belini Rodrigues, Everton Nunes da Silva, André Marques dos Santos, Leonor Maria Pacheco Santos

**Affiliations:** 1 Graduate Program in Collective Health, University of Brasília, Brasília, Federal District, Brazil; 2 Faculty of Ceilândia, University of Brasilia, Brasília, Federal District, Brazil; 3 Company ATsaúde Consultancy, São Paulo, São Paulo, Brazil; 4 Public Health Department, University of Brasília, Brasília, Federal District, Brazil; Universidad Nacional Autonoma de Nicaragua Leon, NICARAGUA

## Abstract

Anencephaly, encephalocele, and spina bifida are congenital neural tube defects and are the main causes of neonatal morbidity and mortality and impose a heavy economic burden on health systems. This study to estimates the direct costs of neural tube defects from the perspective of the Brazilian Ministry of Health, and the prevented cases and cost savings during the period in which mandatory folic acid fortification was in effect in the country (2010–2019). It is a top-down cost-of-illness oriented study based on the prevalence of the disorders in Brazil. Data were collected from the Brazilian Ministry of Health’s outpatient and hospital information system databases. The direct cost was estimated from the total patient-years, allocated by age and type of disorder. Prevented cases and cost savings were determined by the difference in the prevalence of the disorders in the pre- and post-fortification periods based on the total number of births and the sum of outpatient and hospital costs during the period. The total cost of outpatient and hospital services for these disorders totaled R$ 92,530,810.63 (Int$ 40,565,896.81) in 10 years; spina bifida accounted for 84.92% of the total cost. Hospital costs were expressive of all three disorders in the first year of the patient’s life. Between 2010 and 2019, mandatory folic acid fortification prevented 3,499 live births with neural tube defects and resulted in R$ 20,381,586.40 (Int$ 8,935,373.25) in hospital and outpatient cost savings. Flour fortification has proved to be a valuable strategy in preventing pregnancies with neural tube defects. Since its implementation, there has been a 30% decrease in the prevalence of neural tube defects and a 22.81% decrease associated in hospital and outpatient costs.

## Introduction

Congenital anomalies are structural or functional changes that originate in intrauterine life and are a major cause of the global burden disease in low- and middle-income countries [1]. Neural tube defects (NTDs) consist of congenital structural anomalies and are major causes of neonatal morbidity and mortality [2]. Anencephaly, encephalocele, and spina bifida are the most frequent alterations of the central nervous system and result from an incomplete closure of the structures from the brain and spinal cord between the third and fourth week of uterine development [3]. Globally each year, 300,000 newborns suffer from NTDs [1]. Nutritional, environmental, and genetic factors have been identified as risk factors for NTDs [4–6]. An adequate intake of folic acid (FA), either by supplementation or fortification of food, in the periconception period reduces the risk of pregnancies with NTDs [7–9].

Countries that have implemented universal and mandatory FA fortification of foods have shown a significant reduction in the prevalence of NTDs [10,11]. In the last three decades, advances in prenatal diagnostic imaging and neonatal care have significantly improved the survival of children suffering from spina bifida, 75% of whom can reach adulthood [12]. Studies indicate a heavy economic burden of NTDs on health systems, which can be substantially reduced with preventive measures, such as flour fortified with FA [13,14]. Most economic analyses of the cost of these disorders have been conducted in the context of high-income countries, which tend not to reflect the economic burden of NTDs in low- and middle-income countries [15].

In Brazil, an upper-middle-income country, the strategy adopted by the Ministry of Health to reduce iron deficiency anemia and problems related to NTDs has been the fortification of food [16]. The Brazilian Health Regulatory Agency (ANVISA) decided that FA fortification of wheat and corn flours would be mandatory as of June 2004 [17]. The country recorded a decrease in the prevalence of NTDs of approximately 30%, from 0.79/1000 in the pre-fortification period (2001–2004) to 0.55/1000 in the post-fortification period (2005–2014) [18]. However, there is a lack of economic analyses that describe the economic burden of NTDs on the country or the potential cost savings attributed to the mandatory FA fortification after 2004. Cost-of-illness studies are relevant because they measure the total costs of a given disease and estimate the savings attributable to disease elimination, and are also useful for cost–benefit analyses of preventive interventions [19]. This type of economic analysis serves as a guide and relevant resource to develop policies and prioritize and manage public health [20].

This study estimated: the direct healthcare costs of NTDs from the perspective of Brazil’s Unified Health System (SUS) at the federal level, and the prevented cases and cost savings during the period in which the mandatory FA fortification of wheat and corn flours was in effect in Brazil (2010–2019).

## Methods

### Study design

From the perspective of the Brazilian SUS at the federal level of the Ministry of Health, this study applies a top-down approach to examine the cost of NTDs (cost of illness) based on prevalence. The estimated direct healthcare costs were stratified by type of NTD (anencephaly, spina bifida, and encephalocele), age, and year. The period from January 2010 to December 2019 was analyzed.

### Data source

In Brazil, SUS constitutes the national health system and is governed by the principles of universality, equity, and integrality [21]. With 5,570 municipalities, the public health system benefits approximately 200 million Brazilians annually, thus totaling approximately 2.8 billion outpatient and hospital services in basic and specialized medical care [22]. Moreover, SUS provides epidemiological, sanitary, and environmental surveillance actions and services, pharmaceutical care, urgent and emergency care, and primary health care. Organizing health care in SUS is the responsibility of the three levels of the Brazilian Federation: the Union, States, and municipalities [23]. The computerization of SUS activities is consolidated at the national level and is made publicly available by the SUS Information Technology Department (DATASUS, acronym in Portuguese) of the Ministry of Health [24].

In this study, we used the databases of the Outpatient (SIASUS) and Hospital (SIHSUS) Information Systems. We adopted the coding of the International Classification of Diseases (ICD-10) to extract cost data, stratified by year and age in the 2010–2019 period: Q00 (Anencephaly and similar malformations), Q01 (Encephalocele), and Q05 (Spina bifida) [25]. This procedure allowed us to ascertain the number of procedures performed and their respective amounts reimbursed by the Ministry of Health based on the amounts provided in the Table of Procedures, Medication, Orthotics/Prosthetics, and Special Materials of the Unified Health System (SIGTAP, acronym in Portuguese). The descriptive chart (S1 Table) exhibits the fields used in these databases.

### Study variables

Ordinance 321/2007 of the Ministry of Health prescribes the SIGTAP table [26], which contains the expenses for outpatient and hospital procedures. The amount for hospitalizations corresponds to the following: I) Hospital Services, including daily rates, room charges, food, hygiene, support staff for patients occupying beds, materials, and medication; and II) Professional Services, which corresponds to the fraction of professional aid (physicians, dental surgeons, and obstetric nurses) involved in hospitalizations. Outpatient Services include the cost of professional services, materials, and medication [27].

### Population

To estimate the number of patients with NTDs in the outpatient system, we referred to individual reports, home care, psychosocial care, and miscellaneous reports as there was no unique patient code in the outpatient database. These databases were unified to account for the number of patients in the national health care field, which is available in each of the databases. We grouped the data by year, ICD-10, and age.

The hospital system does not provide a field with a unique code to calculate the number of patients, so we created a code by gathering the information available in the fields for sex, date of birth, and municipality and postal code of residence. With this new code, we calculated the number of patients and grouped them by year, ICD-10, and age.

The study population was stratified into the following age groups to calculate costs: < 1 year, 1 year, 2–5 years, 6–10 years, 11–20 years, 21–30 years, 31–40 years, 41–50 years, 51–60 years, 61–70 years, 71–80 years, and > 81 years.

### Estimating NTD rates

To estimate the number of prevented cases owing to mandatory fortification during the 2010–2019 period, we utilized the following information: I. the rates of prevalence of the disorders in the pre-fortification (2001–2004) and post-fortification (2005–2014) periods, as provided by Santos et al. (2016) [18]; II. the number of births in the research period (2010–2019), which totaled 29,157,184 live births and 312,516 stillbirths according to a 2021 epidemiological report by the Ministry of Health [28] based on the Live Birth Information System (SINASC, acronym in Portuguese). In this ten-year period, 13,443 live births with NTDs were registered: 52% (*n = 7*,036) were live births with spina bifida, 33% (*n* = 4,397) were live births with anencephaly, and 15% (*n* = 2,010) were live births with encephalocele.

Example of the calculation:

Estimate of prevented ICD-10 cases Q05 = (pre-fortification prevalence rate x total births in the period)–(post-fortification prevalence rate x total live births in the period)

### Estimating medical cost savings

We estimated the outpatient and hospital cost savings attributable to fortification based on the difference between the prevalence of NTDs in the pre- and post-fortification periods, as provided by Santos et al. [18], and applied it to the sum of the outpatient and hospital costs for each type of NTD during the 2010–2019 period. We performed this calculation as per the following example:

Estimate of the cost saving for ICD-10 Q05 = outpatient costs + hospital costs in post- fortification period (2010–2019) x the difference in prevalence (0.23 live births) of the disorder between the pre- and post-fortification periods

### Statistical analysis

We determined the total and average costs for the period (2010–2019). The total cost of each NTD included outpatient procedures and hospitalizations throughout the period (2010–2019). We calculated the average cost dividing the total cost by the number of years in the period (10 years), stratified by the type of NTD. The values were adjusted for inflation for each year based on the national consumer price index (IPCA, acronym in Portuguese) [29]. The adjusted values reflect prices in December 2019. In addition to stratification by disease, costs were also stratified by age group. We also estimated cost variations throughout the period using 2010 as the base year. Thus, values lower than one indicate that there was a cost reduction relative to 2010. Conversely, values greater than one suggest that there was a cost increase relative to 2010.

### Health care and purchasing power parities

An alternative method to the exchange rate, purchasing power parity (PPP) [30], accounts for the differences in income and cost of living in relation to the international dollar. We converted the national currency (R$) into international dollars (Int$) using the purchasing power parity conversion factor: 2.281 for the year 2019 [31].

The project was submitted to the Ethics Committee of the School of Health Sciences at the University of Brasília and approved under number 4.192.532 in August 2020.

## Results

In the 2010–2019 period, SIASUS recorded 365,892 authorizations for outpatient procedures to treat NTDs: spina bifida accounted for 97.30% (n = 356,005), encephalocele for 2.63% (n = 9,641), and anencephaly for 0.07% (n = 246). The most frequent outpatient procedures were physical therapy and physical rehabilitation services for encephalocele and spina bifida for all ages (see S2 Table).

In the same period, the hospital information system registered 13,129 authorizations for hospitalization: spina bifida corresponded to 78.75% (n = 10,339), encephalocele to 14.51% (n = 1,905), and anencephaly to 6.74% (n = 885). The most frequent procedures were surgeries of the nervous system and corrections of malformations (see S3 Table). The < 1 year age group represented approximately 70% of hospitalizations for the treatment of all types of NTDs. At this age, the length of stay for spina bifida, encephalocele, and anencephaly was six, seven, and three days, respectively (see S4 Table).

Between 2010 and 2019, we found a total cost of R$ 78.58 million (Int$ 34,452,075.09) for treatment of spina bifida in the public health system, including both outpatient care (43.99%) and hospitalizations (56.01%). In general, the economic burden of spina bifida is more pronounced in the early age groups (up to 20 years), thus representing 90.74% of the total cost of the disease in the period under analysis. However, we detected striking differences between the costs based on the type of care. For example, hospital costs are strongly concentrated in the population up to the age of 1 year (86.47%), while outpatient costs are more prevalent in the age groups between 2 and 20 years (73.68%) (Table 1).

**Table 1:** Total and average costs of outpatient and hospital procedures for spina bifida in Brazil in the 2010–2019 period, stratified by age group.

Age Group	^1^Total Outpatient Costs [A] R$	^*2*^ *PPC* *Dollar* *Int$*	%	^3^Total Hospital Costs [B] R$	*PPC* *Dollar* *Int$*	%	Total Costs [A+B] R$	*PPC* *Dollar* *Int$*	Annual Average Period R$	*PPC* *Dollar* *Int$*
<1	1,016,416.00	445,601.05	2,94	36,733,097.06	16,103,944.34	83,47	37,749,513.05	16,549.545,39	3,774,951.31	1,654,954.54
1	2,419,812.87	1,060,856.14	7,00	889,205.05	389,831.23	2,02	3,309,017.92	1,450.687,38	330,901.79	145,068.73
2–5	9,124,285.35	4,000,125.09	26,39	1,957,449.86	858,154.25	4,45	11,081,735.21	4,858.279,35	1,108,173.52	485,827.93
6–10	7,871,893.94	3,451,071.43	22,77	1,419,556.32	622,339.46	3,23	9,291,450.26	4,073.410,89	929,145.03	407,341.09
11–20	8,483,228.61	3,719,083.12	24,54	1,396,579.61	612,266.37	3,17	9,879,808.22	4,331.349,50	987,980.82	433,134.94
21–30	2,418,036.22	1,060,077.25	6,99	473,803.80	207,717.58	1,08	2,891,840.02	1,267.794,83	289,184.00	126,779.48
31–40	1,253,199.08	549,372.67	3,62	196,693.73	86,231.35	0,45	1,449,892.81	635,639.11	144,989.28	63,563.91
41–50	740,817.14	324,777.35	2,14	239,463.05	104,981.60	0,54	980,280.20	429,758.96	98,028.02	42,975.89
51–60	642,550.18	281,696.70	1,86	282,036.18	123,645.84	0,64	924,586.36	405,342.55	92,458.64	40,534.25
61–70	415,708.29	182,248.26	1,20	296,742.78	130,093.28	0,67	712,451.08	312,341.55	71,245.11	31,234.15
71–80	131,141.71	57,493.07	0,38	94,368.06	41,371.35	0,21	225,509.77	98,864.43	22,550.98	9,886.44
>81	59,643.39	26,147.91	0,17	29,455.00	12,913.19	0,07	89,098.39	39,061.10	8,909.84	3,906.11
**Total**	**34,576,732.79**	*15*,*158*,*585*.*17*	**100**	**44,008,450.50**	*19*,*293*,*489*.*91*	**100**	**78,585,183.29**	*34*,*452*,*075*.*09*	**7,858,518.33**	*3*,*445*,*207*.*50*

Note: The costs refer to amounts reimbursed by the Ministry of Health to providers based on the SIGTAP table. The amounts were adjusted for inflation for each year, according to the IPCA, and represent prices in December 2019.

1. Outpatient costs comprise appointments for specialized care; biochemical tests and imaging; orthotics, prosthetics, and mobility aids; and allowances for travel, accommodation, and food expenses for patients/accompanying persons relative to an estimated average of 7,533 patients seen annually between 2010 and 2019.

2. Amount in R$ divided by the conversion factor (2.281) adjusted by PPP (Int$) for the year 2019.

3. Hospitalization costs encompass daily rates, room charges, hospital materials, medication, ancillary tests and therapies, surgeries, and professional services relative to an estimated average of 869 patients seen per year between 2010–2019.

The economic burden of encephalocele was 6.5 times lower than that of spina bifida, totaling R$ 11.94 million (Int$ 5,235,427.08) in the 2010–2019 period. Hospital services play an essential role in the care of patients with encephalocele, which corresponded to 95.15% of the total cost of this disorder in the public health system. Unlike hospital costs, which are essentially concentrated in patients up to the age of one year (86.51%), outpatient costs are more evenly distributed among the age groups (Table 2).

**Table 2:** Total and average costs of outpatient and hospital procedures for encephalocele in Brazil in the 2010–2019 period, stratified by age group.

Age Group	^1^Total Outpatient Costs[A] R$	^*2*^ *PPC* *Dollar* *Int$*	%	^3^Total Hospital Costs[B] R$	*PPC* *Dollar* *Int$*	%	Total Costs [A+B] R$	*PPC* *Dollar* *Int$*	Annual Average Period R$	*PPC* *Dollar* *Int$*
<1	50,895.75	22,312.91	8,80	9,831,244.75	4,310,059.07	86,51	9,882,140.49	4,332,371.98	988,214.05	433,237.19
1	34,973.79	15,332.65	6,05	332,378.65	145,716.19	2,92	367,352.44	161,048.85	36,735.24	16,104.88
2–5	68,747.12	30,139.02	11,89	334,907.89	146,825.02	2,95	403,655.01	176,964.05	40,365.50	17,696.40
6–10	64,803.88	28,410.29	11,21	394,191.83	172,815.35	3,47	458,995.71	201,225.65	45,899.57	20,122.56
11–20	77,486.11	33,970.23	13,40	287,793.19	126,169.74	2,53	365,279.30	160,139.98	36,527.93	16,013.99
21–30	61,924.44	27,147.93	10,71	33,000.48	14,467.54	0,29	94,924.92	41,615.48	9,492.49	4,161.54
31–40	51,865.22	22,737.93	8,97	24,562.25	10,768.19	0,22	76,427.47	33,506.12	7,642.75	3,350.61
41–50	59,731.11	26,186.37	10,33	68,988.37	30,244.79	0,61	128,719.47	56,431.15	12,871.95	5,643.11
51–60	53,946.18	23,650.23	9,33	27,967.89	12,261.24	0,25	81,914.07	35,911.47	8,191.41	3,591.14
61–70	28,899.66	12,669.73	5,00	19,269.19	8,447.69	0,17	48,168.85	21,117.42	4,816.88	2,111.74
71–80	16,704.57	7,323.35	2,89	3,291.41	1,442.96	0,03	19,995.97	8,766.31	1,999.60	876.633
>81	8,334.83	3,654.02	1,44	6,100.63	2,674.54	0,05	14,435.46	6,328.56	1,443.55	632.85
**Total**	**578,312.65**	*253*,*534*.*69*	**100**	**11,363,696.52**	*4*,*981*,*892*.*38*	**100**	**11,942,009.17**	*5*,*235*,*427*.*08*	**1,194,200.92**	*523*,*542*.*70*

Note: The costs refer to amounts reimbursed by the Ministry of Health to providers based on the SIGTAP table. The amounts were adjusted for inflation for each year, according to the IPCA, and represent prices in December 2019.

1. Outpatient costs comprise appointments for specialized care; biochemical tests and imaging; orthotics, prosthetics, and mobility aids; and allowances for travel and food expenses for patients/accompanying persons relative to an estimated number of 430 patients in the period.

2. Amount in R$ divided by the conversion factor (2.281) adjusted by PPP (Int$) for the year 2019.

3. Hospitalization costs encompass daily rates, room charges, hospital materials, medication, ancillary tests and therapies, surgeries, and professional services relative to an estimated number of 172 patients in the period.

Anencephaly is the most severe condition of NTDs; death occurs soon after birth. These low life expectancy these individuals’ is reflected in the economic burden, which is the lowest among the NTDs. Anencephaly totaled R$ 2.00 million (Int$ 878,394.87) over 10 years (2010–2019), essentially in hospital costs (99.39%) (Table 3).

**Table 3:** Total and average costs of outpatient and hospital procedures for anencephaly in Brazil in the 2010–2019 period.

Age Group	^1^Total Outpatient Costs[A] R$	^*2*^ *PPC* *Dollar* *Int$*	%	^3^Total Hospital Costs[B] R$	*PPC* *Dollar* *Int$*	%	Total Costs [A+B] R$	*PPC* *Dollar* *Int$*	Annual Average Period R$	*PPC* *Dollar* *Int$*
<1	12,128.08	5,317.00	100	1,991,490.62	873,077.86	100	2,003,618.70	878,394.87	200,361.87	87,839.48
**Total**	**12,128.08**	*5*,*317*.*00*	**100**	**1,991,490.62**	*873*,*077*.*86*	**100**	**2,003,618.70**	*878*,*394*.*87*	**200,361.87**	*87*,*839*.*48*

Note: The costs refer to amounts reimbursed by the Ministry of Health to providers based on the SIGTAP table. The amounts were adjusted for inflation for each year, according to the IPCA, and represent prices in December 2019.

1. Outpatient costs comprise appointments for specialized care, biochemical tests and imaging, orthotics and prosthetics, and allowance for travel and food expenses for patients/accompanying persons relative to an estimated number of eight patients in the period.

2. Amount in R$ divided by the conversion factor (2.281) adjusted by PPP (Int$) for the year 2019.

3. Hospitalization costs encompass anesthesia, surgical and clinical treatments, biochemical tests and imaging, and hospital and medical services relative to an estimated number of 87 patients in the period.

4. The rare cases of anencephaly recorded in the outpatient and hospital information systems in age groups over one year of age, which were likely due to recording errors, were not analyzed.

**Fig 1:**
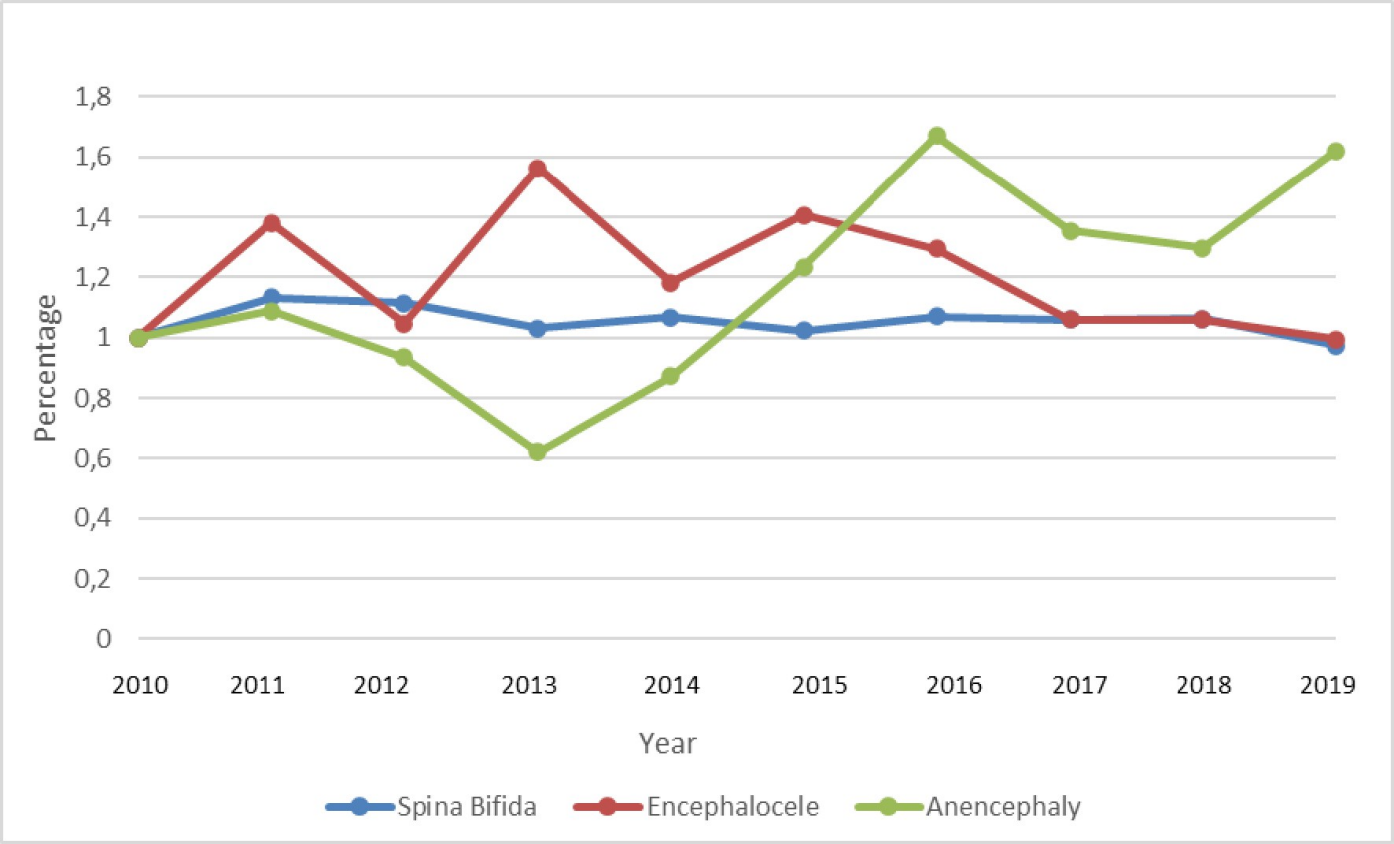
Annual variation of the total cost of outpatient and hospital procedures for neural tube defects (spina bifida, encephalocele, and anencephaly) in Brazil from 2010–2019. Note: The costs refer to amounts reimbursed by the Ministry of Health to providers based on the SIGTAP table. The amounts were adjusted for inflation for each year, according to the IPCA, and represent prices in December 2019.

The variation in the cost of NTDs exhibited a distinct trend throughout the period (2010–2019). The cost of spina bifida demonstrated greater stability, remaining practically constant throughout the period. The total cost of encephalocele showed spiked increases in the years 2011 (40%), 2013 (60%), and 2015 (40%), relative to the base year (2010). However, in the last three years of the period (2017–2019), total costs remained very close to the value verified in the base year. The cost of anencephaly, in turn, dropped sharply in 2013 (40%) and rose more than 60% in 2016 and in 2019, relative to the base year (Fig 1).

The results presented in Table 4 suggest that the mandatory folic acid fortification of flour would have prevented 3,499 cases of NTDs over the course of 10 years (2010–2019) in Brazil, with spina bifida accounting for 50% of these cases. If we consider all births instead of only live births, the number of prevented cases would increase to 7,367 in the same period, in which spina bifida represents 28% of the cases. Furthermore, fortification would be responsible for reducing the cost of NTDs by R$ 20.38 million (Int$ 8.935.373,25) in the 2010–2019 period (Table 4).

**Table 4:** Prevented cases and cost savings due to folic acid fortification in Brazil from 2010–2019.

**Type of neural tube defect**	**Pre-fortification**		**Post-fortification**		**Estimated** **Prevented Cases**
	Prevalence (%)	No. of cases (n)	Prevalence (%)	No. of cases (n)	
**Live Births** ^**3**^					
Anencephaly	0,21	6.123	0,16	4.665	1.458
Encephalocele	0,08	2.333	0,07	2.041	292
Spina Bifida	0,28	8.164	0,22	6.415	1.749
**Total 3.499**					
**All Births** ^**3**^					
Anencephaly	0,42	12.377	0,26	7.662	4.715
Encephalocele	0,09	2.652	0,07	2.063	589
Spina Bifida	0,30	8.841	0,23	6.778	2.063
**Total 7.367**					

	**Verified outpatient and hospital costs** **(R$)**	^**1**^ **Difference in prevalence** **(%)**	**Counterfactual outpatient and hospital costs** **(R$)**	**Estimated** **cost savings**
**Live Births**				
Anencephaly	2,003,618.70	24,2	2,488,494.42	- 484,875.72
Encephalocele	11,942,009.17	14,6	13,685,542.50	- 1,743,533.34
Spina Bifida	78,585,183.29	23,6	96,738,360.63	- 18,153,177.34
**Total**				- 20,381,586.40
**All Births**				
Anencephaly	2,003,618.70	38,2	2,771,004.66	- 767,385.96
Encephalocele	11.942,009.17	13,7	13,578,064.42	- 1,636,055.26
Spina Bifida	78.585,183.29	23,8	97,288,456.91	- 18,703,273.62
**Total**				**- 21,106,714.84**

Note: The costs refer to amounts reimbursed by the Ministry of Health to providers based on the SIGTAP table. The amounts were adjusted for inflation for each year, according to the IPCA, and represent prices in December 2019.

1. According to Santos et al. (2016), differences in prevalence between pre- and post-fortification, by type of neural tube defect and births.

2. Based on Santos et al. (2016), the rates of prevalence between the pre- and post-fortification periods.

3. Based on Epidemiological Bulletin n° 6, congenital anomalies in Brazil, 2010 to 2019: 29,157,184 live births and 29,469,700 all births.

## Discussion

This study’s contribution to the literature is its estimation of the economic burden of neural defects in an upper-middle-income Latin American country’ with a population of over 200 million inhabitants. The total cost of these disorders, including outpatient and hospital services, amounted to R$ 92,530,810.63 (Int$ 40,565,896.81) over 10 years (2010–2019). Spina bifida accounted for 84.92% of the total cost, corresponding to R$ 78,585,183.29 (Int$ 34,452,075.09), over the same period. Hospital costs constituted the majority for the three disorders, especially in the first year of the patient’s life. Outpatient costs were significant only for spina bifida, representing 43.99% of the total cost of this disorder. Considering live births, our results suggest that the mandatory folic acid fortification of flour would have prevented 3,499 cases of NTDs and saved R$ 20,381,586.40 (Int$ 8,935,373.25) in hospital and outpatient costs in the country between 2010 and 2019. Although spina bifida represented 50% of the prevented cases of NTDs in the period, its representation was more substantial with respect to cost savings (89.06%).

Other studies have also estimated the costs of NTDs on a global level, which tend to align with our results. To make use of a common monetary unit, the values extracted from the studies were converted by PPP to adjust for the countries’ cost of living. The total hospital costs for patients with spina bifida in the first year of life were Int$ 1.6 million in Brazil (536 cases, 2019), Int$ 1 million in Chile [32] (130 cases, 2001), Int$ 9.8 million in South Africa (946 cases, 2006) [33], and Int$ 74 million in the United States (1,133 cases, 2003) [34]. A comparison of these results should be made with caution as the studies reflect aspects specific to their countries (relative prices, availability of health care, and health financing), to different methodological decisions (type of study, included costs, and data quality), and to the data collection period (year of the study). In our study, the variation in the total cost of NTDs is attributable to the higher number of cases recorded during the period (2010–2019) and is not related to the cost of the services. According to the Live Births Information System (SINASC) [28], the highest recorded number of cases of congenital anomalies was observed in 2016 (17,662) due to two factors: (i) an increase in cases of microcephaly in the country and (ii) changes made in 2011 in declaring live births, which made it possible to notify a greater number of anomalies per child and thereby improve the detection of cases in the following years [28].

We also verified that the costs and type of health services changed based on age and the type of NTD; our results were consistent with clinical experience [35,36]. In the first year of life (< 1 year), hospital costs represented the highest percentage of total costs for the treatment of NTDs due to frequent surgeries to close the defect and treat hydrocephalus in this age group (see appendix 1B). Similarly, Colombo et al. (2013) [37] found that neurosurgeries were frequent in the first year of life and that most were performed to close the defect and place the ventriculoperitoneal shunt to treat hydrocephalus. Another study evidenced that the ventriculoperitoneal shunt was placed before the age of 6 months in 93% of the cases of open spina bifida [38].

The US [39–41] and European [37,42] studies also identified the highest costs in the interval between 0 and 1 year. Hospitalizations accounted for most of the medical expenses in the first year of life and decreased in the other age groups. These results corroborate our findings. A systematic review by Yi et al. (2011) [13] included 14 cost-of-illness studies and demonstrated that the direct lifetime costs for people with NTDs are substantial and that most of their expenses are due to hospitalizations in childhood and comorbidities in adulthood.

A review including a meta-analysis [43] indicated the factors that may have contributed to the improved survival of live births with spina bifida in the last 30 years: (i) precision of prenatal diagnosis; (ii) increase in terminations for the most severe types of fetal anomalies; (iii) advances in neonatal and surgical care, including early neonatal or fetal surgery for spinal repair; and (iii) periconceptional folic acid intake or fortification likely reduced the number of severe types of spina bifida. Consequently, there were more adults than newborns and children with spina bifida in the United States in 2019 [12]. Adults with spina bifida continue to require neurosurgical care [44], outpatient services, and periodic inpatient hospitalization [45].

Unlike the US studies, our results indicated children and adolescents as the age groups that most often used outpatient and hospital services for spina bifida in Brazil. In this group, the most frequent procedures were physical therapy (60%) and physical rehabilitation (25%), followed by orthopedic orthotics and assistive mobility devices (10%), all resulting in substantial outpatient costs. Our findings corroborate results presented by Bamer et al. (2010) [46], who verified a high percentage regarding the use and cost of orthotics and prosthetics among individuals with spina bifida between the ages of 0 and 15 years. An Italian study [37] indicated that orthotics and cases of open spina bifida, and non-ambulatory individuals, were the most significant components of the direct costs.

There was a lower frequency of orthotic procedures and use of assistive mobility devices among adults and older adults with spina bifida, which resulte in lower outpatient costs. These findings indicate that this component may have had a possible interference in outpatient costs. Individuals with spina bifida throughout their lives undergo physical therapy due to motor impairment and use orthotics to stabilize their joints and prevent deformities, in addition to using crutches, canes, walkers, or wheelchairs to support their mobility [47].

As for encephalocele, outpatient costs were high in the adult age group, and there was a higher frequency of physical therapy (80%) and imaging exams in the period (20%) (see S2 Table). No cost studies respect to encephalocele were found in the literature. Nonetheless, a Canadian study demonstrated that adult patients with complex physical disabilities used outpatient procedures (96.5%) more often than inpatient hospital services (3.5%). It concluded that these patients with complex physical disabilities since childhood had ongoing health problems and required frequent care [45].

Regarding NTD prevention strategies, the folic acid fortification of foods has helped reduce prevalence in underserved populations but has not eliminated it [48]. In Brazil, mandatory FA fortification reduced cases of NTDs by around 30%, thereby resulting in an estimated 350 prevented cases of live births with NTDs each year and savings in the amount of R$ 2 million [Int$ 893,537.32 (2019)] each year for SUS due owing to the averted costs of hospitalizations and outpatient procedures. In comparison with other countries, we found that Chile reduced the prevalence of NTDs by 43% [49] (109 prevented cases of spina bifida) and saved an estimated Int$ 2.5 million (2001) due to the direct costs averted [32]. The prevalence of NTDs decreased by 41% in South Africa (406 prevented cases of spina bifida) [33], which generated ZAR 40.6 million [Int$ 6 million (2007)] in annual savings. The prevalence of spina bifida decreased by 47% in the United States (767 prevented cases of live births), amounting to USD $ 319 million in annual savings in 2014 [50].

The discrepancy in cost savings between countries arises from differences in their respective health systems, time frames, and the types of costs included in the economic analyses. Only the US study considered the lifetime medical and non-medical costs for individuals with spina bifida. In the case of Brazil, the direct (medical) costs for the three types of NTDs over a 10-year period were included herein. The meager savings may be explained by the lag in the financial values of hospital and outpatient procedures. In SUS, all procedures are assigned financial values in a single table (SIGTAP), and studies [51,52] reveal that these values are not adequately updated. This table serves as a reference for the reimbursement of services, but it does not necessarily correspond to the actual amount spent to fund relatively and highly complex services. There are other criteria for allocating budgetary resources to municipalities and states so that they may fund services in the region [53]. In this study, these values were not included in the total cost of the disorders.

A systematic review [15] of 13 cost-effectiveness studies of the mandatory FA fortification of flour found that FA dosages above 0.30 mg/100 g had a higher cost-effectiveness ratio and provided greater benefits (prevented cases and direct cost savings). This systematic review indicated a median return ratio of 17.5:1. In other words, for every monetary unit spent on the mandatory fortification program, there would be a return of 17.5 monetary units. In Brazil, however, it was not possible to perform a cost–benefit analysis of the mandatory fortification, because the flour milling industry does not disclose the cost of the flour enrichment process.

This study had some limitations. First, the direct costs of NTDs incurred in the private healthcare insurance sector were not included in the total cost of the disorder, which may have generated underestimated results. The SIHSUS/SIASUS databases cover approximately 75% of Brazilian health services [54]. Second, it was not possible to perform per capita analyses, as the registration units were not individualized and the expenses were aggregated in the procedure and hospitalization authorization documents used to bill the services provided [55]. This document may present more than one procedure or hospitalization on record for the same person in situations where they return for appointments or new hospitalizations within a short period of time [56]. Third, the lack of reliability of the SIHSUS data regarding secondary diagnoses due to the absence of information in medical records and problems inherent to the international coding for the disorders may have influenced our conclusions [57]. Despite these limitations, the study exhibits considerable strengths: (i) it includes data on a large sample of people with spina bifida and encephalocele; (ii) it presents estimated costs for different age groups, thus providing a point estimation of the various components applied in health care for users with NTDs; and (iii) the databases used provide relevant and comprehensive information about the economic burden associated with spina bifida and encephalocele on SUS.

### Implications for public policies and the health system

In Brazil, more than 55.4% of pregnancies are unplanned [58], and adherence to the use of FA supplements is very low both in the periconception period (4.3%) and during pregnancy (31.8%) [59]. Fortified flour has proven to be a valuable strategy for preventing pregnancies with NTDs. Since the implementation of FA fortification program, there has been a 30% reduction in the prevalence of NTDs and a 22.81% reduction in hospital and outpatient costs for NTD patients. This means that mandatory FA fortification constitutes a national public health strategy that contributes to reducing the burden of the disorder on Brazilian society. Accordingly, the country is aligned with the Sustainable Development Goals of the 2030 Agenda [60], which focuses on improving nutritional status and reducing inequalities among populations worldwide. Finally, we expect efforts dedicated to enhance the primary prevention of NTDs and provide adequate health actions and services to individuals with NTDs, especially easily accessible neurology services in all regions of Brazil [53,61].

## Supporting information

S1 Table Shows the bases and fields used for filters in database. (PDF)

S2 Table Frequency of outpatient procedures by type of neural tube defect and age group in the period 2010–2019, Brazil. (PDF)

S3 Table Frequency of hospital procedures by type of neural tube defect and age group in the period 2010–2019, Brazil. (PDF)

S4 Table Mean length of hospitalization by type of neural tube defect and age group in the period 2010–2019, Brazil. (PDF)

## References

[1] World Health Organization. Congenital anomalies 2022 Feb 28. [cited 20 May 2022] In World Health Oranization [internet]. Available from: https://www.who.int/news-room/fact-sheets/detail/birth-defects.

[2] World Health Organization. Prevention of neural tube defects. In Integrated Management of Pregnancy and Childbirth (IMPAC). 2006 [cited 25 May 2022]. Available from: www.who.int/reproductivehealth/publications/maternal_perinatal_health.htm.

[3] Padmanabhan R., Etiology pathogenesis and prevention of neural tube defects. Congenit Anom (Kyoto). 2006 Jun;46(2):55–67. doi: 10.1111/j.1741-4520.2006.00104.x 16732763

[4] Li K, Wahlqvist ML, Li D. Nutrition, One-Carbon Metabolism and Neural Tube Defects: A Review. Nutrients. 2016 Nov 23;8(11):741. doi: 10.3390/nu8110741 27886045 PMC5133124

[5] Chen CP. Syndromes, disorders and maternal risk factors associated with neural tube defects (V). Taiwan J Obstet Gynecol. 2008 Sep;47(3):259–66. doi: 10.1016/S1028-4559(08)60122-9 18935987

[6] Seidahmed MZ, Miqdad AM, Al-Dohami HS, Shareefi OM. A case of fetal valproate syndrome with new features expanding the phenotype. Saudi Med J. 2009 Feb;30(2):288–91. 19198722

[7] Berti C, Fekete K, Dullemeijer C, Trovato M, Souverein OW, Cavelaars A, Dhonukshe-Rutten R, et al. Folate intake and markers of folate status in women of reproductive age, pregnant and lactating women: a meta-analysis. J Nutr Metab. 2012; 2012:470656. doi: 10.1155/2012/470656 23024859 PMC3449134

[8] De-Regil LM, Fernández-Gaxiola AC, Dowswell T, Peña-Rosas JP. Effects and safety of periconceptional folate supplementation for preventing birth defects. Cochrane Database Syst Rev. 2010 Oct 6;(10):CD007950. doi: 10.1002/14651858.CD007950.pub2 Update in: Cochrane Database Syst Rev. 2015;12:CD007950. 20927767 PMC4160020

[9] Castillo-Lancellotti C, Tur JA, Uauy R. Impact of folic acid fortification of flour on neural tube defects: a systematic review. Public Health Nutr. 2013 May;16(5):901–11. doi: 10.1017/S1368980012003576 22850218 PMC10271422

[10] Crider KS, Bailey LB, Berry RJ. Folic acid food fortification-its history, effect, concerns, and future directions. Nutrients. 2011 Mar;3(3):370–84. doi: 10.3390/nu3030370 22254102 PMC3257747

[11] Food Fortification Initiative. Enhancing Grains for Healthier Lives. Country profiles 2018. [cited 2022 May 5]. Database: Food Fortification Initiative [internet] Available from: http://www.ffinetwork.org/country_profiles/index.php.

[12] Kiehna EN, Blount JP, McClung Smith C, Ocal E, Chatterjee S. Introduction. Advancing the care of children with spina bifida, prenatally and postnatally. Neurosurg Focus. 2019 Oct 1;47(4): e1. doi: 10.3171/2019.8.FOCUS19666 31574463

[13] Yi Y, Lindemann M, Colligs A, Snowball C. Economic burden of neural tube defects and impact of prevention with folic acid: a literature review. Eur J Pediatr. 2011 Nov;170(11):1391–400. doi: 10.1007/s00431-011-1492-8 21594574 PMC3197907

[14] Saing S, Haywood P, van der Linden N, Manipis K, Meshcheriakova E, Goodall S. Real-World Cost Effectiveness of Mandatory Folic Acid Fortification of Bread-Making Flour in Australia. Appl Health Econ Health Policy. 2019 Apr;17(2):243–254. doi: 10.1007/s40258-018-00454-3 30617458

[15] Rodrigues VB, Silva END, Santos MLP. Cost-effectiveness of mandatory folic acid fortification of flours in prevention of neural tube defects: A systematic review. PLoS One. 2021 Oct 21;16(10):e0258488. doi: 10.1371/journal.pone.0258488 34673787 PMC8530293

[16] Brazil. National Health Surveillance Agency. Report on the Monitoring of the Fortification of Wheat and Corn with Iron and Folic Acid. 2020; 1:31. Available from: https://www.gov.br/anvisa/pt-br/centraisdeconteudo/publicacoes/fiscalizacao-e-monitoramento/programas-nacionais-de-monitoramento-de-alimentos/relatorio-fortificacao-de-farinhas-2019-sem-marcas-retificacao.pdf.

[17] Brazil. National Health Surveillance Agency. Resolution of the Collegiate Board n° 344 of December 13, 2002. Approves the technical regulation for the fortification of wheat flour and corn flour with iron and folic acid. 2002 Dec 13. Available from: http://www.anvisa.gov.br/legis/resol/2002/344_02rdc.htm.

[18] Santos LM, Lecca RC, Cortez-Escalante JJ, Sanchez MN, Rodrigues HG. Prevention of neural tube defects by the fortification of flour with folic acid: a population-based retrospective study in Brazil. Bull World Health Organ. 2016 Jan 1;94(1):22–9. doi: 10.2471/BLT.14.151365 26769993 PMC4709794

[19] Oliveira ML, Santos LMP, Silva EN. Methodological bases for cost-of-illness studies in Brazil. Journal of Nutrition. 2014; 27:585–595.

[20] Jo C. Cost-of-illness studies: concepts, scopes, and methods. Clin Mol Hepatol. 2014 Dec;20(4):327–37. doi: 10.3350/cmh.2014.20.4.327 25548737 PMC4278062

[21] Brazil. Ministry of Health. Executive Secretary. Unified Health System (SUS): principles and achievements. 1st ed. Ministry of Health; 2000.

[22] Giovanella L, Mendoza-Ruiz A, Pilar ACA, Rosa MC, Martins GB, Santos IS, et al. Universal health system and universal coverage: unraveling assumptions and strategies. Report in Public Health. 2018; 23:1763–1776. 10.1590/1413-81232018236.05562018 29972485

[23] Brazil. Ministry of Health. Unified Health System (SUS): structure, principles and how it works. 2020 nov 14 and updated 2021dec 31. Available from: https://www.gov.br/saude/pt-br/assuntos/saude-de-a-a-z/s/sus-estrutura-principios-e-como-funciona.

[24] Brazil. Ministry of Health. Database of the Unified Health System-DATASUS. [cited 2022 May 03]. Database Datasus. Available from: http://www.datasus.gov.br.

[25] Wells RHC, Bay-Nielsen H, Braun R, Israel RA, Laurenti R, Maguin P, Taylor E. ICD-10: international statistical classification of diseases and health-related problems. 2011; [cited 2022 May. 01] Available from: https://apps.who.int/iris/handle/10665/42980?locale-attribute=es.

[26] Brazil. Ministry of Health. DATASUS. System for Management of the Table of Procedures, Medicines, Orthoses, Prostheses and Special Materials (SIGTAP, OPM) of SUS. 2020. [cited 2022 April 01]. Database Datasus. Available from: http://sigtap.datasus.gov.br/tabela-unificada/app/sec/inicio.jsp.

[27] Brazil. Ministry of Health. Secretariat of Health Care. Department of Regulation, Evaluation and Control. Systems of Health Care Information: Historical Contexts, Advances and Prospects in SUS/Pan American Health Organization. 2015. Available from: http://www.escoladesaude.pr.gov.br/arquivos/File/sistemas_informacao_atencao_saude_contextos_historicos.pdf.

[28] Brazil. Ministry of Health. General Coordination of Information and Epidemiological Analysis of the Department of Health Analysis and Surveillance of Noncommunicable Diseases. Epidemiological Bulletin. Congenital anomalies in Brazil, 2010 to 2019: analysis of a priority group for birth surveillance. 2021; 1–22. Available from: https://www.gov.br/saude/pt-br/centrais-de-conteudo/publicacoes/boletins/boletins-epidemiologicos/edicoes/2021/boletim_epidemiologico_svs_6_anomalias.pdf.

[29] Brazilian Institute of Geography and Statistics. National system of consumer price indexes. Complete table of historical series; 2022 [cited 2022 Jan 03]. Database IBGE. Available from: https://www.ibge.gov.br/estatisticas/economicas/precos-e-custos/9256-indice-nacional-de-precos-ao-consumidoramplo.html?edicao=20932&t=series-historicas.

[30] De Souza JL. Dollar PPC. In The magazine of information and debates of the Institute for Economic and Applied Research, 2008. Year n°5 issue 40. [cited 2022 April 04] Available from: https://www.ipea.gov.br/portal/index.php?option=com_content&view=article&id=3198.

[31] The World Bank. World Development Indicators database, PPP conversion; 2022. Database worldbank [cited 2022 Jun 05] Available from: https://data.worldbank.org/indicator/PA.NUS.PPP?locations=BR.

[32] Llanos A, Hertrampf E, Cortes F, Pardo A, Grosse SD, Uauy R. Cost-effectiveness of a folic acid fortification program in Chile. Health Policy. 2007 Oct;83(2–3):295–303. 511 doi: 10.1016/j.healthpol.2007.01.011 17363103

[33] Sayed AR, Bourne D, Pattinson R, Nixon J, Henderson B. Decline in the prevalence of neural tube defects following folic acid fortification and its cost-benefit in South Africa. Birth 515 Defects Res A Clin Mol Teratol. 2008 Apr;82(4):211–6. doi: 10.1002/bdra.20442 18338391

[34] Arth AC, Tinker SC, Simeone RM, Ailes EC, Cragan JD, Grosse SD. Inpatient Hospitalization Costs Associated with Birth Defects Among Persons of All Ages, United States, 2013. MMWR Morb Mortal Wkly Rep 2017; 66:41–46. 10.15585/mmwr.mm6602a1. PMC5657658 28103210

[35] Stoll C, Dott B, Alembik Y, Roth MP. Associated malformations among infants with neural tube defects. Am J Med Genet A. 2011 Mar;155ª(3):565–8. doi: 10.1002/ajmg.a.33886 21337695

[36] Chance A, Sandberg DI. Hydrocephalus in patients with closed neural tube defects. Childs Nerv Syst. 2015 Feb;31(2):329–32. doi: 10.1007/s00381-014-2492-6 25028246

[37] Colombo GL, Di Matteo S, Vinci M, Gatti C, Pascali MP, De Gennaro M, et al. A cost-of-illness study of spina bifida in Italy. Clinicoecon Outcomes Res. 2013 Jul 2; 5:309–16. doi: 10.2147/CEOR.S42841 23861590 PMC3704355

[38] Johnson KL, Dudgeon B, Kuehn C, Walker W. Assistive technology use among adolescents and young adults with spina bifida. Am J Public Health. 2007 Feb;97(2):330–6. doi: 10.2105/AJPH.2004.050955 17194874 PMC1781409

[39] Waitzman NJ, Romano PS, Scheffler RM. Estimates of the economic costs of birth defects. Inquiry. 1994 Summer; 31(2):188–205. 8021024

[40] Waitzman N, Romano P, Grosse S. The Halflife of Cost-of-Illness Estimates: The Case of Spina Bifida. Salt Lake City, UT: Department of Economics, University of Utah; 2004. Working Paper Series.

[41] Ouyang L, Grosse SD, Armour BS, Waitzman NJ. Health care expenditures of children and adults with spina bifida in a privately insured U.S. population. Birth Defects Res A Clin Mol Teratol. 2007 Jul;79(7):552–8. doi: 10.1002/bdra.20360 17335056

[42] Bowles D, Wasiak R, Kissner M, van Nooten F, Engel S, Linder R, et al. Economic burden of neural tube defects in Germany. Public Health. 2014 Mar;128(3):274–81. doi: 10.1016/j.puhe.2013.12.001 24559770

[43] Glinianaia SV, Morris JK, Best KE, Santoro M, Coi A, Armaroli A, Rankin J. Long-term survival of children born with congenital anomalies: A systematic review and meta-analysis of population-based studies. PLoS Med. 2020 Sep 28;17(9):e1003356. doi: 10.1371/journal.pmed.1003356 32986711 PMC7521740

[44] Piatt JH Jr. Adults with myelomeningocele and other forms of spinal dysraphism: hospital care in the United States since the turn of the millennium. J Neurosurg Spine. 2016 Jul;25(1):69–77. doi: 10.3171/2015.9.SPINE15771 26926705

[45] Young NL, Steele C, Fehlings D, Jutai J, Olmsted N, Williams JI. Use of health care among adults with chronic and complex physical disabilities of childhood. Disabil Rehabil. 2005 Dec 15;27(23):1455–60. doi: 10.1080/00222930500218946 16523542

[46] Bamer AM, Connell FA, Dudgeon BJ, Johnson KL. Frequency of purchase and associated costs of assistive technology for Washington State Medicaid program enrollees with spina bifida by age. Disabil Health J. 2010 Jul;3(3):155–61. doi: 10.1016/j.dhjo.2009.10.009 21122780

[47] Brea CM, Munakomi S. Spina Bifida. [Updated 2022 Feb 9]. In: StatPearls Treasure Island (FL): StatPearls Publishing; 2022. Available from: https://www.ncbi.nlm.nih.gov/books/NBK559265/. 32644691

[48] Neto MC. Prevention of open neural tube defects: NTD. 2st ed. São Paulo: Brazilian Federation of Gynecology and Obstetrics Associations; 2020.

[49] Hertrampf E, Cortés F. Folic acid fortification of wheat flour: Chile. Nutr Rev. 2004 Jun;62(6 Pt 2):S44–8; discussion S49. doi: 10.1111/j.1753-4887.2004.tb00074.x 2004.tb00074.x. 15298448

[50] Grosse SD, Berry RJ, Mick Tilford J, Kucik JE, Waitzman NJ. Retrospective Assessment of Cost Savings From Prevention: Folic Acid Fortification and Spina Bifida in the U.S. Am J Prev Med. 2016 May;50(5 Suppl 1):S74–S80. doi: 10.1016/j.amepre.2015.10.012 26790341 PMC4841731

[51] França A e Giustina APD. Out-of-date management system of the unified table of procedures (SIGTAP) Unified Health System. 2016; 1–14. [cited 2022 May10]. Available from: http://www.uniedu.sed.sc.gov.br/wp-content/uploads/2016/10/ARTUR-FRAN%C3%87A.pdf.

[52] Federal Council of Medicine. The defasation in the SUS table affects most hospital procedures. CFM. 2015; 1–3. [cited 2022 May10]. Available from: http://portal.cfm.org.br/index.php?option=com_content&view=article&id=25491:defasagem-na-tabelasus-afeta-maioria-dos-procedimentos-hospitalares&catid.

[53] Brazil. Office of the Comptroller General (CGU). Institute of Applied Economic Research. Council for Monitoring and Evaluation of Public Policies. Health Care of the Population for Procedures in Medium and High Complexity (MAC) 2019; 1:40. (cited 2022 May 18). Available from: https://www.gov.br/economia/pt-br/acesso-ainformacao/participacao-social/conselhos-e-orgaoscolegiados/cmap/.

[54] Brazil. Ministry of Health. The Brazilian experience in health information systems Ministry of Health. 1st ed. Publisher of the Ministry of Health; 2009.

[55] Lucena, CDRX. Descriptive analysis of elective admissions in 2012 and the use of the National Health Card (CNS) in the Authorization for Hospital Admission (AIH) as a strategy for qualification of health information. University of Brasília. 2014. Available from: https://repositorio.unb.br/handle/10482/19736.

[56] Melo, Thamires Francelino Mendonça de et al. Direct costs of prematurity and factors associated with birth and maternal conditions. (Journal of Public Health. 2022, v. 56–49. Epub 13 June 2022. ISSN 1518-8787. doi: 10.11606/s1518-8787.2022056003657 35703603 PMC9239337

[57] Bittencourt SDA, Camacho LAB, Leal MDC. The Hospital Information System and its application to public health. Reports in Public Health. 2006; 22:19–30. 16470279 10.1590/s0102-311x2006000100003

[58] Theme-Filha MM, Baldisserotto ML, Fraga AC, Ayers S, da Gama SG, Leal MD. Factors associated with unintended pregnancy in Brazil: cross-sectional results from the Birth in Brazil National Survey, 2011/2012. Reprod Health. 2016 Oct 17;13(Suppl 3):118. doi: 10.1186/s12978-016-0227-8 27766945 PMC5073899

[59] Mezzomo CLS, Garcias GL, Sclowitz ML, Sclowitz IT, Brum CB, Fontana T, Unfried RI. Prevention of neural tube defects: prevalence of folic acid supplementation during pregnancy and associated factors in Pelotas, Rio Grande do Sul State, Brazil. Reports in Public Health. 2007,23;11: 2716–2726. 17952264 10.1590/s0102-311x2007001100019

[60] Transforming our world: the 2030 Agenda for Sustainable Development Adopted at the United Nations Sustainable Development Summit on 25 September 2015. Available from: https://www.un.org/ga/search/view_doc.asp?symbol=A/RES/70/1&Lang=E.

[61] Yacob A, Carr CJ, Foote J, Scullen T, Werner C, Mathkour M, et al. The Global Burden of Neural Tube Defects and Disparities in Neurosurgical Care. World Neurosurg. 2021 May;149:e803–e820. doi: 10.1016/j.wneu.2021.01.096 Epub 2021 Feb 1. . 33540098

