# Supplementary material for: Prevented cases of neural tube defects and cost savings after folic acid fortification of flour in Brazil
Source: PLoS One. 2023 Feb 22;18(2):e0281077. doi: 10.1371/journal.pone.0281077 (PMC9946232; doi:10.1371/journal.pone.0281077)
Supplement: S1 Table — (PDF) [file pone.0281077.s001.pdf]

Table 1 shows the bases used, as well as the fields used for filters. In these fields the ICDs Q00 (Anencephaly and similar malformations), Q01 (Encephalocele), and Q05 (Spina bifida) were filtered.

Table 1. Description of the databases used

SYSTEM	DESCRIPTION	FILENAME	FIELD
SIASUS	Individual Bulletin	BIUFaamm.dbc	CIDPRI
SIASUS	Outpatient Production	PAUFaamm.dbc	PA_CIDPRI
SIASUS	HOME CARE	SADUFaamm.dbc	CIDPRI
SIASUS	RAAS – PSICOSSOCIAL	PSUFaamm.dbc	CIDPRI
SIASUS	VARIOUS REPORTS	ADUFaamm.dbc	AP_CIDPRI
SIHSUS	Reduced AIH	RDUFaamm.dbc	DIAG_PR
