# Supplementary material for: Prevented cases of neural tube defects and cost savings after folic acid fortification of flour in Brazil
Source: PLoS One. 2023 Feb 22;18(2):e0281077. doi: 10.1371/journal.pone.0281077 (PMC9946232; doi:10.1371/journal.pone.0281077)
Supplement: S2 Table — (PDF) [file pone.0281077.s002.pdf]

Table 2. Frequency of Outpatient Procedures by type of neural tube defect and age group in the period 2010-2019.

Age Group	Hospital Procedures	N	%
	**SPINA BIFIDA**		
<1	Accompaniment in physical rehabilitation	8.482	**41**
	Physiotherapy care in neurokinetic-functional disorders	6.249	**30**
	Physiotherapy care in the pre and post-operative period	2.364	**12**
	Image Examinations	2.000	**10**
	Orthopedic orthoses and locomotion aids	700	**3**
	Consultation with higher level professionals in specialized care	300	**1**
	Travel allowance/food for patient and accompanying person	250	**1**
	Others	204	**1**
	Total	20.549	100
1-10	Accompaniment in physical rehabilitation	137.602	**59**
	Physiotherapeutic care in neurokinetic-functional disorders	45.149	**19**
	Orthopedic orthoses and locomotion aids	23.750	**10**
	Image exams	9.754	**4**
	Physiotherapy care in the pre and post-operative period	9.632	**4**
	Others	9.014	**4**
	Total	234.901	100
11-20	Accompaniment in physical rehabilitation	20.000	**36**
	Physiotherapeutic care in neurokinetic-functional disorders	13.000	**23**
	Orthopedic orthoses and locomotion aids	11.000	**20**
	Image exams	4.100	**7**
	Physiotherapy care in the pre and post-operative period	3.900	**7**
	Others	3.871	**7**
	Total	55.871	100
	Physiotherapy care in the pre- and post-operative periods	5.000	**26**
	Accompaniment physical rehabilitation	4.735	**25**

	Orthopedic orthoses and locomotion aids	4.000	**21**
	Physiotherapy care in neurokinetic-functional disorders	2.500	**13**
21-40	Image Examinations	1.500	**8**
	Others	1.391	**7**
	Total	19.126	100
	Physiotherapy care in the pre and postoperative period	13.053	**73**
	Physiotherapy care in neurokinetic-functional disorders	1.719	**9**
	Follow-up in physical rehabilitation	1.416	**8**
	Orthopedic orthoses and locomotion aids	845	**5**
41-60	Image Examinations	611	**3**
	Others	485	**2**
	Total	18.000	100
	Physiotherapy care in the pre and postoperative period	4.802	**68**
	Physiotherapy care in neurokinetic-functional disorders	862	**12**
	Follow-up in physical rehabilitation	627	**9**
61-80	Orthopedic orthoses and locomotion aids	465	**7**
	Others	288	**4**
	Total	7.044	100
	Physiotherapy care in the pre and postoperative period	242	**47**
	Physiotherapy care in neurokinetic-functional disorders	155	**30**
	Follow-up in physical rehabilitation	41	**8**
>81	Orthopedic orthoses and locomotion aids	62	**12**
	Other	14	**3**
	Total	514	100
	**ENCEPHALOCELE**		
	Physiotherapeutic care in neurokinetic-functional disorders	384	**65**
	Imaging exams	117	**20**
<1	Others	40	**15**
	Total	895	100
1-10	Physiotherapeutic care in neurokinetic-functional disorders	3.000	**78**

	Imaging exams	450	**12**
	Others	393	**10**
	Total	3.843	100
11-20	Physiotherapeutic care in neurokinetic-functional disorders	806	**78**
	Imaging exams	168	**16**
	Others	59	**6**
	Total	1.033	100
21-40	Physiotherapy care in neurokinetic-functional disorders	976	**70**
	Image Examinations	272	**20**
	Travel allowance/food	75	**5**
	Others	67	**5**
	Total	1.390	100
41-60	Physiotherapeutic care in neurokinetic-functional disorders	1.190	**73**
	Imaging exams	388	**24**
	Others	49	**3**
	Total	1.627	100
61-80	Physiotherapeutic care in neurokinetic-functional disorders	491	**69**
	Imaging exams	197	**28**
	Others	26	**4**
	Total	714	100
>81	Physiotherapeutic care in neurokinetic-functional disorders	114	**82**
	Imaging exams	25	**18**
	Total	139	100
	**ANENCEPHALY AND OTHER SIMILAR MALFORMATIONS**		
<1	Home care by a multi-professional team	120	**45**
	Urgent care in specialized care	59	**22**

Image Examinations	63	**24**
Others	25	**9**
Total	267	100
