# Supplementary material for: Prevented cases of neural tube defects and cost savings after folic acid fortification of flour in Brazil
Source: PLoS One. 2023 Feb 22;18(2):e0281077. doi: 10.1371/journal.pone.0281077 (PMC9946232; doi:10.1371/journal.pone.0281077)
Supplement: S3 Table — (PDF) [file pone.0281077.s003.pdf]

Table 3. Frequency of Hospital Procedures by type of neural tube defect and age group in the period 2010-2019.

Age Group	Hospital Procedures	N	(%)
	**SPINA BIFIDA**		
<1	Surgical Treatment for Spinal Malformation	2806	**45**
	Surgical treatment for hydrocephalus (shunt)	1426	**23**
	Clinical treatment of Spina Bifida	1230	**20**
	Clinical treatment of congenital deformities of the Osteomuscular System	418	**7**
	Others	320	**5**
	Total	6198	100
01-10	Clinical Treatment of Spina Bifida	2087	**76**
	Surgical treatment of spinal malformation	266	**10**
	Surgical treatment of hydrocephalus (shunt)	215	**8**
	Others	179	**7**
	Total	2747	100
11-20	Clinical Treatment of Spina Bifida	674	**76**
	Surgical treatment of spinal malformation	107	**12**
	Surgical treatment of hydrocephalus (shunt)	81	**9**
	Others	28	**3**
	Total	890	100
21-40	Clinical Treatment of Spina Bifida	58	**21**
	Surgical treatment of hydrocephalus (shunt)	56	**20**
	Surgical treatment of lower limb fasciotomy	55	**20**
	Surgical treatment of spinal malformation	38	**14**
	Surgical treatment of hip dislocation	36	**13**
	Others	34	**12**
	Total	277	100
41-60	Surgical treatment of hydrocephalus (shunt)	64	**50**
	Surgical treatment of lower limb fasciotomy	17	**13**

	Surgical treatment of hip dislocation	14	**11**
	Surgical treatment of spinal malformation	12	**9**
	Clinical treatment of Spina Bifida	11	**9**
	Others	10	**8**
	Total	128	100
61-80	Surgical treatment of hydrocephalus (shunt)	58	**66**
	Surgical treatment of lower limb fasciotomy	10	**11**
	Surgical treatment of hip dislocation	9	**10**
	Surgical/Medical Emergency Treatment	6	**7**
	Others	5	**6**
	Total	88	100
>81	Surgical treatment of hydrocephalus (ventricular shunt)	4	**36**
	Surgical treatment of hip dislocation	4	**36**
	Surgical Treatment of Lower Limb Fasciotomy	2	**18**
	Others	1	**9**
	Total	11	100
	**ENCEPHALOCELE**		
<1	Surgery for correction of the malformation	713	**47**
	Surgical treatment of congenital anomalies of the Nervous System	414	**27**
	Surgical treatment of congenital malformation of the Osteomuscular System	263	**17**
	Craniofacial surgery for cranio and maxillofacial anomalies	56	**4**
	Pediatric Emergency Care	40	**3**
	Others	35	**2**
	Total	1521	100
1-10	Surgery for correction of the malformation	68	**28**
	Surgical treatment of congenital anomalies of the Nervous System	57	**24**
	Surgical treatment of congenital malformations of the Osteomuscular System	39	**16**

	Craniofacial surgery for cranio and buccomaxillofacial anomalies	30	**12**
	Pediatric and surgical emergency care	25	**10**
	Others	22	**9**
	Total	241	100
11-20	Surgical treatment of congenital anomalies of the Nervous System	22	**32**
	Surgical treatment of congenital malformations of the Osteomuscular System	19	**28**
	Dental treatment for patients with special needs	10	**15**
	Surgery for correction of spinal malformation	9	**13**
	Others	8	**12**
	Total	68	100
21-40	Dental treatment for patients with special needs	20	**59**
	Urgent care in the surgical/medical clinic	5	**15**
	Surgical treatment of congenital anomalies of the nervous system	4	**12**
	Surgery for correction of spinal malformation	3	**9**
	Others	2	**6**
	Total	34	100
41- 60	Surgical treatment of congenital anomalies of the nervous system	12	**40**
	Dental treatment for patients with special needs	6	**20**
	Surgical treatment of congenital malformations of the Osteomuscular System	5	**17**
	Craniofacial surgery for cranial and maxillofacial anomalies	4	**13**
	Others	3	**10**
	Total	30	100
61-80	Surgical treatment of congenital anomalies of the Nervous System	2	**20**
	Surgical treatment of congenital malformations of the Osteomuscular System	2	**20**
	Urgent care in surgical/medical clinic	2	**20**

	Dental treatment for patients with special needs	2	**20**
	Others	2	**20**
	Total	10	100
>81	Surgical treatment of congenital anomalies of the Nervous System	1	**100**
	Total	1	100
	**ANENCEPHALY AND OTHER SIMILAR MALFORMATIONS**		
<1	Surgical treatment of congenital anomalies of the Nervous System	610	**69**
	Surgical treatment of congenital malformations of the Osteomuscular System	211	**24**
	Pediatric Emergency Care	38	**4**
	Others	26	**3**
	Total	885	100
