# Supplementary material for: Prevented cases of neural tube defects and cost savings after folic acid fortification of flour in Brazil
Source: PLoS One. 2023 Feb 22;18(2):e0281077. doi: 10.1371/journal.pone.0281077 (PMC9946232; doi:10.1371/journal.pone.0281077)
Supplement: S4 Table — (PDF) [file pone.0281077.s004.pdf]

Table 4: Mean length of hospitalization by type of neural tube defect and age group in the period 2010-2019, Brazil

ENCEPHALOCELE																							
Year	2010		2011		2012		2013		2014		2015		2016		2017		2018		2019		Total		Mean	%
Age group	Total AIH	Nº of day	Total AIH	Nº of day	Total AIH	Nº of day	Total AIH	Nº of day	Total AIH	Nº of day	Total AIH	Nº of day	Total AIH	Nº of day	Total AIH	Nº of day	Total AIH	Nº of day	Total AIH	Nº of day	AIH	Total of day		
<1	559	4703	633	5060	633	5536	674	5453	734	5628	783	5175	827	4235	827	4235	794	4274	755	4032	7219	48331	6,7	63,20
1	48	454	44	371	54	631	45	754	62	717	60	541	41	456	41	456	50	407	37	404	482	5191	10,8	4,22
2_5	100	1173	137	1333	140	1407	112	1079	115	1129	125	935	92	877	92	877	80	808	68	428	1061	10046	9,5	9,29
6_10	107	959	156	1376	140	1328	96	867	103	835	94	953	79	682	79	682	68	708	70	539	992	8929	9,0	8,68
11_20	87	873	127	1310	123	1129	87	885	106	979	117	955	85	855	85	855	74	1045	57	519	948	9405	9,9	8,30
21_30	18	98	19	147	17	111	32	335	23	111	28	245	35	165	35	165	34	222	42	180	283	1779	6,3	2,48
31_40	7	62	12	46	12	72	11	97	16	122	21	132	17	71	17	71	25	90	22	82	160	845	5,3	1,40
41_50	11	93	5	19	6	57	8	40	12	81	6	35	11	55	11	55	8	27	9	48	87	510	5,9	0,76
51_60	6	51	13	98	9	49	7	37	12	47	9	90	8	93	8	93	5	59	4	25	81	642	7,9	0,71
61_70	8	74	8	34	10	79	6	47	4	26	5	72	9	80	9	80	4	24	4	64	67	580	8,7	0,59
71_80	3	13	4	34	5	26	3	48	4	54	0	0	2	4	2	4	2	33	1	2	26	218	8,4	0,23
>81	1	4	2	23	0	0	3	16	2	20	0	0	3	8	3	8	0	0	2	2	16	81	5,1	0,14
Total																					11422	86557	7,6	100

SPINA BIFIDA

YEAR	2010		2011		2012		2013		2014		2015		2016		2017		2018		2019		Total		Mean	%
Age group	Total AIH	Nº of day	Total AIH	Nº of day	Total AIH	Nº of day	Total AIH	Nº of day	Total AIH	Nº of day	Total AIH	Nº of day	Total AIH	Nº of day	Total AIH	Nº of day	Total AIH	Nº of day	Total AIH	Nº of day	AIH	Total of day		
<1	416	3223	447	3292	509	3752	515	4214	609	4130	707	3826	815	4018	733	2934	709	3001	738	2902	6198	35292	5,7	59,8
1	42	382	38	315	66	578	54	662	64	590	66	355	50	556	41	407	48	343	38	361	507	4549	9,0	4,9
2_5	103	1127	139	1249	150	1347	123	1013	137	1044	147	882	86	724	100	799	74	674	83	539	1142	9398	8,2	11,0
6_10	102	910	168	1350	153	1324	96	786	118	824	103	831	77	587	87	648	70	554	67	421	1041	8235	7,9	10,0
11_20	76	641	131	1261	128	1090	95	799	122	956	139	891	99	687	75	551	59	582	51	355	975	7813	8,0	9,4
21_30	13	87	17	142	13	100	30	320	21	106	18	180	22	174	18	130	14	169	23	118	189	1526	8,1	1,8
31_40	6	61	9	33	8	59	10	96	13	115	9	107	11	113	8	47	8	49	10	51	92	731	7,9	0,9
41_50	9	87	5	19	6	57	7	33	12	81	6	35	6	84	6	41	3	18	3	23	63	478	7,6	0,6
51_60	6	51	12	93	7	31	7	37	11	46	7	65	4	36	6	89	2	31	3	24	65	503	7,7	0,6
61_70	9	74	7	33	9	78	5	37	4	26	5	71	4	22	8	72	4	24	3	60	58	497	8,6	0,6
71_80	3	13	4	34	5	26	2	19	4	54	0	0	2	8	1	3	2	33	1	2	24	192	8,0	0,2
>81	1	4	1	20	0	0	2	13	2	20	0	0	0	0	3	8	0	0	2	2	11	67	6,1	0,1
																				Total	10365	69281	6,68	100

ANENCEPHALY AND OTHER SIMILAR MALFORMATIONS

Year	2010		2011		2012		2013		2014		2015		2016		2017		2018		2019		Total		Mean	%
Age group	Total AIH	Nº of day	Total AIH	Nº of day	Total AIH	Nº of day	Total AIH	Nº of day	Total AIH	Nº of day	Total AIH	Nº of day	Total AIH	Nº of day	Total AIH	Nº of day	Total AIH	Nº of day	Total AIH	Nº of day	AIH	Total of day		
<1	65	234	80	341	72	457	68	223	96	452	93	249	117	444	103	364	98	243	93	339	885	3346	3,7	100

Note: AIH - authorization document for hospitalization
Nº of day - days of hospitalization by age group and year
